# Self-reported oral health and associated factors in the North Finland 1966 birth cohort at the age of 31

**DOI:** 10.1186/1472-6831-14-155

**Published:** 2014-12-16

**Authors:** Terho Lintula, Ville Laitala, Paula Pesonen, Kirsi Sipilä, Marja-Liisa Laitala, Anja Taanila, Vuokko Anttonen

**Affiliations:** Institute of Dentistry, University of Oulu, Oulu, Finland; Institute of Dentistry, University of Eastern Finland, Joensuu, Finland; Oral and Maxillofacial Department, Kuopio University Hospital, Kuopio, Finland; Medical Research Center, Oulu University Hospital and University of Oulu, Oulu, Finland; Institute of Health Sciences, University of Oulu, Oulu, Finland; Primary Health Care Unit, University Hospital of Oulu, Oulu, Finland

**Keywords:** Self-reported oral health, Health behavior, Dental health surveys, Adults, Epidemiological

## Abstract

**Background:**

The Northern Finland 1966 birth cohort (NFBC 1966) is an epidemiological study where the participants have been controlled since pregnancy both in field tests and using questionnaires. This study aimed to evaluate cross-sectionally the association of self-reported oral symptoms (dental caries and bleeding of gums) with sociodemographic and health behavior factors among the subjects.

**Methods:**

Of the 11,541 original members of the cohort, 8,690 (75%) responded to the questionnaire on oral health (dental decay, gingival bleeding and self-estimated dental treatment need) and sociodemographic factors, general health and health behavior. Cross-tabulation and chi-squared tests as well as multiple logistic regression analysis were used to analyze the association between the outcome and explanatory variables.

**Results:**

The study group was equally distributed between the genders. One third of the subjects reported having dental decay, one fourth gingival bleeding and a half a dental treatment need. As compared to women, men reported significantly more frequently symptoms (p < 0.05). Logistic regression analysis revealed low tooth brushing frequency increasing the odds most for all oral symptoms ((OR 1.57 (1.39–1.78) for dental decay, 1.94 (1.68-2.24) for gingival bleeding and 1.42 (1.26-1.61) for dental treatment need). Frequent smoking was associated with dental decay (OR 1.63 (1.44–1.84)) and treatment need OR (1.39 (1.23–1.56)), whereas poor general health (OR 1.71 (1.48–1.96)) and high BMI (OR 1.19 (1.03–1.36)) both were associated with gingival bleeding.

**Conclusions:**

Males with single marital status, BMI over 25, poor general health and poor oral health behaviors are at risk for self-reported poor oral health and dental treatment need.

## Background

Although oral health status in Finland has improved in the past 20 years, oral health related problems are still common. According to the Health 2000 survey, two out of three adults had periodontal problems and every third dental caries. Women had less dental caries and reported brushing their teeth significantly more frequently compared to men. In addition, socioeconomic status and lifestyle, such as smoking and eating habits, were associated with oral health behaviors [1].

Health behaviors have been found to be relevant indicators of oral health status. For example, dietary habits and oral hygiene play a significant role in the pathogenesis of dental decay. Diets rich in fermentable carbohydrates, such as sucrose-containing fizzy drinks and sweets, have been found to be associated with poor oral health, particularly with dental caries [2]. In Finland, sales of sweets increased 17.4% during the period 2000–2009, even if consumption of sugar *per se* did not [3]. In addition, soft drink consumption has remained high in the 2000s [4]. Also periodontal diseases are mainly caused by bacteria in the biofilms - plaque. Thus, good oral hygiene remains essential for oral health.

Unhealthy eating habits are connected to obesity, which is presented by the Body Mass Index (BMI). Association between BMI and oral health has been a focus in many recent studies and the results are partly contradictory. It has been reported that individuals with a higher BMI are more prone to have more dental caries than individuals with a lower BMI [5]. BMI has also been found to be associated with periodontal diseases [6, 7]. In industrialized world patients with a higher socioeconomic status are more likely to have a lower BMI compared to those with a lower status [8].

Patients following a healthy lifestyle also pay more attention to their oral health [9]. Individuals with a lower education level have been shown to possess significantly worse oral health than those with a higher education level. A recent Danish study revealed that dental status among adults, *per se*, was associated to educational background and income and socio-behavioral factors significantly affected dentate health [10]. In the Nordic countries, public healthcare has aimed to provide equal access to healthcare for all the citizens and thereby to reduce the impact of the socioeconomic status. In fact, the gap in the attendance rate at dental healthcare between Finnish adolescents from different socioeconomic backgrounds has declined in recent decades [11]. However, literature reveals also contradictory results: among adults, subjects with a higher socioeconomic status have been found to be more likely regular attendees at oral health services [1, 12, 13].

The North Finland Birth Cohort 1966 study was started in the two northernmost provinces in Finland in the year 1965 when the mothers of the cohort members were pregnant. Data on the individuals born into this cohort was collected since the 24th gestational week. Altogether live-born 12,058 infants i.e. 96.3% of all births during 1966 in that area comprised the cohort at baseline. The original data have been supplemented by data collected with postal questionnaires at the ages of 1, 14 and 31 years and various hospital records and national register data (http://kelo.oulu.fi/NFBC/koho1966/nfbc1966description.htm).

The aim of this study was to analyze the association of self-reported oral health and associated risk indicators among young Finnish adults. The study setting also allows detecting the most remarkable risk markers. Another aim was to investigate the distribution of those markers in detail in this study group. In this study we hypothesize that male gender, single marital status, low education level, poor oral hygiene, poor dental health behavior, and smoking are related or represent odds for poor self-reported oral health.

## Methods

### Study population

The study population comprised the Northern Finland 1966 birth cohort, which is an unselected general population birth cohort including 96.3% of all births in 1966 in the two northern provinces of Finland, Oulu and Lapland (n = 12,058). In 1997–1998, a postal questionnaire was sent to those who were still alive and whose address was known (n = 11,541). Of those people, 8,690 responded to the questionnaire (the response rate was 75%) and agreed to participate in the study. The questionnaire consisted of questions about general and oral health as well as health behavior, such as smoking, drinking and physical activity. Information on person’s gender, marital status and education were also achieved.

### Outcome variables

Oral health was inquired by using the following questions: “*In your opinion, do you have caries lesions in your teeth at the moment?” , “In your opinion, do your gums bleed when your brush your teeth?” , “In your opinion, do you have a healthy mouth without a need of any dental treatment?”* The response options for all these questions were yes/no. Presence of self-reported dental caries lesions, gingival bleeding and negative answer to the last question formed the outcome variables.

### Explanatory variables

The explanatory variables were gender, marital status, education, BMI, general health, tooth brushing frequency, consumption of fizzy drinks, and smoking. Marital status was determined using a question with five options (married, co-habiting, divorced, widow, and single). The options were categorized into two classes, married/co-habiting and the rest (living single). Education was determined by a question with three options (less than 9 years of basic education, basic education of 9 years, and matriculation examination). The options were further categorized into two classes, basic education (9 years or less basic education) and matriculation examination. BMI was calculated according to the self-reported weight and height and categorized into two classes, normal (BMI 25 or less) and overweight or obese (BMI more than 25). General health was inquired with the question “*How would do you evaluate your general health at the moment?*” with five options (excellent, good, moderate, poor, and very poor). The options were further categorized into two classes, at least good health and moderate or poorer.

Tooth brushing was inquired with the question *“How often do you brush your teeth?*” with three options (more frequently than once a day, once a day, less than once a day). The options were further categorized into two classes, brushing at least twice a day and once a day or less. Consumption of fizzy drinks was inquired with the question *“How often do you usually consume the following food items (fizzy drinks), considering the last six months?”* , which had six options (less than once a month or not at all, once or twice a month, once a week, a couple of times a week, almost daily, once a day or more). The options were further categorized into two classes, once a week at most and more frequently than once a week. Smoking was inquired with the question *“Do you currently smoke”* with six options (daily, on five to six days a week, on two to four days a week, on one day a week, occasionally, not at all). The options were further categorized to smokers (smoking at least on one day a week) and non-smokers (occasionally or never).

### Statistical methods

The associations between the explanatory and outcome variables were analyzed by cross-tabulation and the significance of the differences were evaluated using Pearson’s chi-squared tests. Odds ratios (OR) and 95% confidence levels (CI) were estimated using multiple logistic regression analyses. The explanatory variables in the multiple logistic regression analysis were gender, marital status, education, BMI, general health, tooth brushing frequency, consumption of fizzy drinks, and smoking. Goodness of fit of the models was tested by Hosmer & Lemeshow test. P-values < 0.05 were considered statistically significant. Distribution of the subjects was illustrated graphically having dental caries as the outcome variable and smoking, BMI, gender and marital status as explanatory variables. All analyses and figures were executed with SPSS (version 18.0, SPSS, Inc., Chicago, Il, USA).

### Ethical issues

Permission to gather data was obtained from the Ministry of Social Affairs and Health, and the study has been approved by the Ethics Committee of the Northern Ostrobothnia Hospital District. Written informed consent was obtained from all participants.

## Results

The basic characteristics of the study population in this age cohort (all born in 1966) are described in Table 1. The study population was equally distributed between the genders. Among the study group, the majority was married/co-habiting and had at least good general health and one third were overweight or obese (Table 1). About fourty percent (39.3%) reported smoking daily and 36.3% reported not smoking at all. As compared to women, a larger proportion of men were overweight and smokers. The proportion of men who reported to brush their teeth less frequently than once a week was twice as compared to the corresponding proportion of women. Men also reported to have lower education and were slightly more often singles than women. There were no differences in self-reported general health between the genders (Table 1).

**Table 1:** **Description of the study population**

		Male (n = 4,167) %	Female (n = 4,523) %	Total (n = 8,690) %
Marital status	married/co-habiting	68.4	76.4	72.6
	single	31.6	23.6	27.4
Education	basic education	69.5	48.3	58.5
	matriculation examination	30.5	51.7	41.5
BMI	optimum weight or below	52.5	73.6	63.4
	overweight or obese	47.5	26.4	36.6
General health	at least good	68.2	68.5	68.4
	moderate or below	31.8	31.5	31.6
Tooth brushing	twice a day	39.5	67.6	54.2
	once a day at most	60.5	32.4	45.8
Smoking	non-smoking	46.9	59.7	53.2
	smokers	53.1	40.3	46.8
Consumption of fizzy drinks	once a week at most	66.1	86.2	76.6
	more than once a week	33.9	13.8	23.4

**Figure 1:**
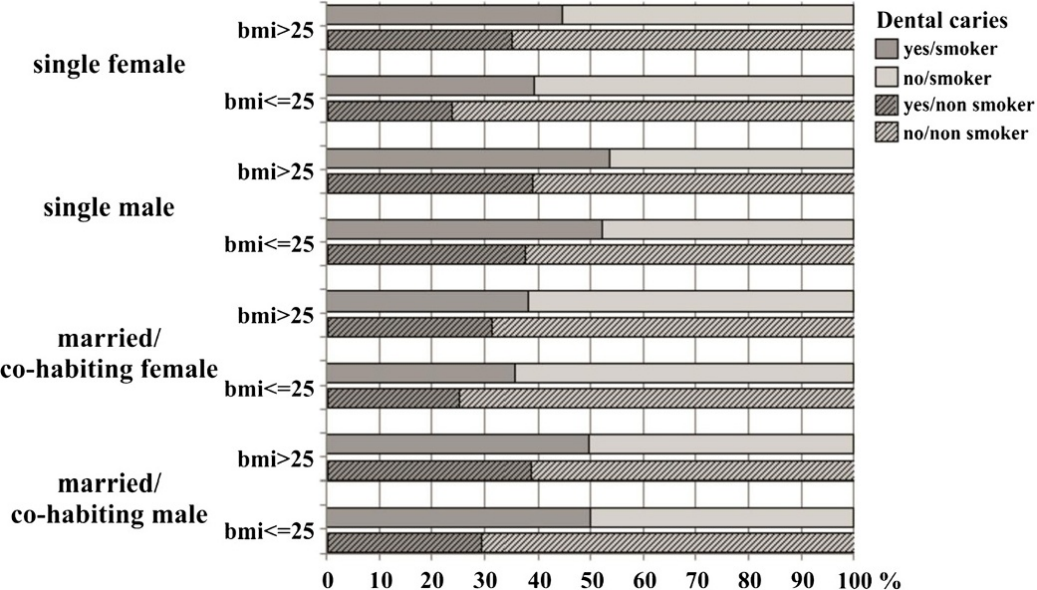
**Distribution of the participants in the study group according to their self-reported dental caries and considering also their smoking habit, BMI, gender and marital status.**

Of the study subjects, 35.2% reported having dental decay, 24.0% bleeding of gums while brushing, and 51.2% reported need of dental treatment. As compared to women, men reported significantly more often dental decay, gingival bleeding and need for dental treatment (Table 2). Being a single male with only basic education, overweight and poor general health were significantly associated with self-reported dental decay, gingival bleeding and need of dental treatment (Table 2). Health behavior was also related to oral health, i.e. consumption of fizzy drinks more often than once a week and brushing frequency of less than twice a day were significantly associated with dental decay, gingival bleeding and need of dental treatment. The smokers reported to have more dental decay and need for restorative dental treatment compared to the non-smokers (Table 2) considering also gender, BMI and marital status (Figure 1). Both males and females despite the marital status with high BMI (>25) tended to report more dental caries than the ones with low BMI (Figure 1).

**Table 2:** **Association of sociodemographic background factors and health behavior with reported oral health in 8,690 subjects included in the Northern Finland 1966 birth cohort**

		Self-reported dental decay		Self-reported gingival bleeding		Self-reported need of dental treatment	
Gender	male (*n* = 4,167)	1,661	41.3%	1,087	27.2%	2,225	56.1%
	female (*n* = 4,523)	1,313	29.7%	929	21.1%	2,034	46.8%
Marital status	married/co-habiting (*n* = 6,252)	2,078	34.0%	1,412	23.2%	3,019	50.2%
	living alone (*n* = 2,364)	873	38.3%	591	26.1%	1,212	53.8%
Education	basic education (*n* = 5,045)	2,013	41.2%	1,275	26.3%	2,668	55.8%
	matriculation examination (*n* = 3,581)	951	27.0%	733	20.9%	1,574	45.0%
BMI	optimum weight or below (*n* = 5,354)	1,694	32.5%	1,117	21.5%	2,518	48.9%
	at least overweight (*n* = 3,090)	1,204	40.1%	846	28.3%	1,619	54.8%
General health	at least good (*n* = 5,901)	1,848	32.1%	1,176	20.5%	2,680	47.1%
	moderate or below (*n* = 2,732)	1,120	42.1%	834	31.5%	1,570	60.3%
Tooth brushing	twice a day (*n* = 4,459)	1,241	28.3%	767	17.5%	1,965	45.3%
	once a day at most (*n* = 3,764)	1,586	43.1%	1,160	31.7%	2,090	57.8%
Consumption of fizzy drinks	once a week at most (*n* = 6,593)	2,122	33.0%	1,460	22.8%	3,156	49.7%
	more than once a week (*n* = 2,016)	836	42.6%	552	28.2%	1,084	56.1%
Smoking	non-smoking (*n* = 2,939)	900	31.4%	714	25.0%	1,373	48.5%
	smokers (*n* = 2,584)	1,180	47.0%	601	24.1%	1,478	60.2%

Association between self-reported oral health with all indicators and background factors were statistically significant (p-value < 0.05) in all except between smoking and reported gingival bleeding.

The logistic regression analyses indicated a strong association of low tooth brushing frequency, poor general health and moderate or severe overweight (BMI ≥ 25) with gingival bleeding (Table 3). Furthermore high smoking frequency and low tooth brushing frequency indicated elevated odds for self-reported need of dental treatment as well as prevalence of dental decay. Low educational status was also associated with elevated odds with prevalence of dental decay. Male gender, single marital status, poor general health and consumption of fizzy drinks more often than once a week increased the odds of gingival bleeding, dental decay, and need of dental treatment (Table 3).

**Table 3:** **Odds ratios and 95% confidence intervals for reported oral health and various factors in 8,690 subjects included in the Northern Finland 1966 birth cohort**

	Self-reported dental decay R (95% CI)	Self-reported gingival bleeding OR (95% CI)	Reported need of dental treatment OR (95% CI)
Gender (male)	1.25 (1.09–1.42)	1.11 (0.96–1.29)	1.14 (1.01–1.29)
Marital status (living alone)	1.16 (1.01–1.32)	1.15 (1.00–1.34)	1.06 (0.93–1.21)
Education (basic education)	1.42 (1.25–1.62)	0.96 (0.83–1.11)	1.23 (1.09–1.39)
BMI (at least overweight)	1.09 (0.96–1.24)	1.19 (1.03–1.36)	1.02 (0.90–1.15)
General health (moderate or below)	1.24 (1.09–1.41)	1.71 (1.48–1.96)	1.51 (1.33–1.70)
Tooth brushing (once a day at most)	1.57 (1.39–1.78)	1.94 (1.68–2.24)	1.42 (1.26–1.61)
Fizzy drinks (more than once a week)	1.22 (1.06–1.39)	1.16 (1.00–1.35)	1.11 (0.97–1.27)
Smoking (smoking a couple days week or more)	1.63 (1.44–1.84)	0.77 (0.67–0.89)	1.39 (1.23–1.56)

Hosmer & Lemeshow *х* ^*2*^ = 9.55, df = 8, p = .298 *х* ^*2*^ = 6.38, df = 8, p = .604 *х* ^*2*^ = 9.08, df = 8, p = .335 goodness of fit test.

## Discussion and conclusions

Men in the NFBC 1966 report significantly more often than women oral health problems and treatment need. Co-habiting or married marital status, high education and good general health appear to be protecting factors against self-reported dental decay, gingival bleeding and treatment need. As obvious, low tooth brushing frequency and poor general health are associated with all three outcome variables. The association between frequent consumption of fizzy drinks and dental decay is well established; interestingly this association is also found between fizzy drinks and gingival bleeding here.

The association between socioeconomic status and oral health is well established [12, 14], which is in accordance with our results even if socioeconomic status here refers to education. In Finland education and consequently profession indicate person’s socioeconomic status [15] health. In this study, marital status (married/co-habiting) and high education level were protective factors against dental decay. Both of these factors may reflect in socially related health behavior. Higher education gives also knowledge and ability to seek and implement information in everyday behavior.

All the risk factors studied here, excluding poor general health, were more prevalent among men than women. A higher proportion of men than women were single, had lower education, were overweight, smoked, and consumed fizzy drinks. They also reported brushing their teeth less frequently compared to women. Consequently, the sociodemographic and health behavior risk factors of poor oral health may concentrate on male gender, who also reported more often than women dental decay, gingival bleeding and dental treatment need. Our results are in accordance with earlier studies [1, 16], which report poorer oral health and behavior among men and boys than women and girls. Both men and women have equal health education at school and equal opportunities for healthcare. Therefore, reasons for the gender differences can only be speculated – perhaps the values associated with health are different among the genders. The possible mechanisms explaining these gender differences should be a topic of future studies.

Oral hygiene in Finnish population has been reported to be among the poorest in Europe when evaluated by the brushing frequency, even if some improvement has been seen lately [17]. In our study, 54% of the study population reported brushing their teeth at least twice a day (40% for men and 68% for women). In a Scottish health survey, 71% of the study population reported brushing their teeth at least twice a day [18]. The low brushing frequency among the NFBC 1966 subjects is surprising because they have received education for oral health promotion since their first years at school (Public Health Act 1972). Because tooth brushing and use of fluoride toothpaste are the factors that have led to improvement in dental caries prevalence, it can be assumed that low brushing frequency has at least partly led to cessation in improvement of dental caries prevalence.

In this study, smoking among the study population was quite common: about 40% of the respondents reported daily smoking. Smoking is also more common among those with a low socioeconomic status [19]. Smoking affects general health but is also associated with poor oral health. In our study, smoking was inversely associated with gingival bleeding. It has been shown that there is an association between periodontal disease and smoking. It is known that smoking may mask the symptoms i.e. gingival bleeding of periodontal disease [20]. This is supported by our findings. Due to the limitation of our study that the subjects were not examined clinically, the association between smoking and dental caries and perceived dental treatment need should be further investigated.

Our results regarding the association between high BMI (>25) and self-reported gingival bleeding are in accordance with those by Saxlin et al. (2011) and Modéer et al. (2011) [6, 7]. Poor dietary habits influence BMI and could raise the caries prevalence. However, this association could not be established here. Modéer et al. (2011) have also reported that obese people brush their teeth less frequently, which in turn could increase the risk of gingival bleeding as well as dental decay, as also shown in our study [6]. In our study, a lower educational status was associated with the increased risk of dental decay, which could be explained by unfavorable dietary habits, as suggested by Lallukka et al. (2006) [14]. Association between dietary habits and oral health *per se* could not be established in our study, as the only question on dietary habits regarded the consumption of fizzy drinks.

The strength of this study is its large, representative study population of young adults, allowing analyses of all the factors presented in the study. All participants were born in 1966, thus being in their early thirties in 1997–1998. The study population is also evenly distributed between the genders. However, the outcome variables are based on self-reporting and not on clinical examination, which is a limitation of the study. Therefore, our results may have been distorted by over- and underestimation of oral health. For example, 23% of the study subjects reported gingival bleeding when brushing their teeth, but in a national study (Health 2000) of the same age group in the early 2000s using clinical examinations [1] more than 69% of 35-44-year-old men and more than 52% of women had gingival disease (at least one periodontal pocket 4 mm or deeper). Therefore, the self-reported gingival bleeding may under-estimate the actual manifestation of the disease. However, in our study group the proportion of those reporting dental decay (34%) is close to that reported in the Health 2000 study in adult population [1] (31%) based on a clinical examination.

In the most recent epidemiologic Oral Health 2011 study [21], 29.2% of males and 14.8% of females in the age group 30 to 44 years had need for restorative treatment; the respective figures for prevalence of periodontal disease indicated by at least one periodontal pocket 4 mm or deeper were 56.5% and 42.8%. According to these figures, there is slight improvement in the oral health status of young adults in the first decades of this millennium. Since early 2000s all Finnish citizens are entitled to dental care sub vented by the state. A further field study of the North Finland 1966 birth cohort was recently conducted (2012–2013), including clinical oral health examination, which allows further analyzing the progress of oral health as well as validity of the self-reported symptoms compared with clinical findings in future in the NFBC 1966. Also it will be interesting to investigate if the difference between the genders persists despite the services available.

Our hypotheses that male gender, single marital status, low education level, and poor dental health behavior are related to poor oral health were confirmed in the present study. However, the association between smoking and gingival bleeding remained obscure. Patients’ self-care is the key to the oral and general health. In order to empower patients to improve their oral health, the dentist should be able to recognize the oral risk factors on an individual basis as early as possible. At the age of the cohort subjects (31 years at the time of the study), the common risk factors that could potentially be influenced are tooth brushing, smoking and consumption of fizzy drinks. Soft drinks may possess risk not only for general health (obesity), and dental caries, but also for periodontal health. This should be investigated in detail in future among this study group.

## Abbreviations

BMI or Body Mass Index: The body mass index (BMI) is defined as the person's body mass (kg) divided by the square of their height (m^2^) (kg/m^2^).
